# *Pseudomonas syringae* pv. *actinidiae* Draft Genomes Comparison Reveal Strain-Specific Features Involved in Adaptation and Virulence to *Actinidia* Species

**DOI:** 10.1371/journal.pone.0027297

**Published:** 2011-11-23

**Authors:** Simone Marcelletti, Patrizia Ferrante, Milena Petriccione, Giuseppe Firrao, Marco Scortichini

**Affiliations:** 1 Research Centre for Fruit Trees, CRA, Roma, Italy; 2 Research Unit for Fruit Trees, CRA, Caserta, Italy; 3 Department of Agricultural and Environmental Sciences, University of Udine, Udine, Italy; University of Birmingham, United Kingdom

## Abstract

A recent re-emerging bacterial canker disease incited by *Pseudomonas syringae* pv. *actinidiae* (*Psa*) is causing severe economic losses to *Actinidia chinensis* and *A. deliciosa* cultivations in southern Europe, New Zealand, Chile and South Korea. Little is known about the genetic features of this pathovar. We generated genome-wide Illumina sequence data from two *Psa* strains causing outbreaks of bacterial canker on the *A. deliciosa* cv. Hayward in Japan (J-*Psa*, type-strain of the pathovar) and in Italy (I-*Psa*) in 1984 and 1992, respectively as well as from a *Psa* strain (I2-*Psa*) isolated at the beginning of the recent epidemic on *A. chinensis* cv. Hort16A in Italy. All strains were isolated from typical leaf spot symptoms. The phylogenetic relationships revealed that *Psa* is more closely related to *P. s*. pv. *theae* than to *P. avellanae* within genomospecies 8. Comparative genomic analyses revealed both relevant intrapathovar variations and putative pathovar-specific genomic regions in *Psa*. The genomic sequences of J-*Psa* and I-*Psa* were very similar. Conversely, the I2-*Psa* genome encodes four additional effector protein genes, lacks a 50 kb plasmid and the phaseolotoxin gene cluster, *argK-tox* but has acquired a 160 kb plasmid and putative prophage sequences. Several lines of evidence from the analysis of the genome sequences support the hypothesis that this strain did not evolve from the *Psa* population that caused the epidemics in 1984–1992 in Japan and Italy but rather is the product of a recent independent evolution of the pathovar *actinidiae* for infecting *Actinidia* spp. All *Psa* strains share the genetic potential for copper resistance, antibiotic detoxification, high affinity iron acquisition and detoxification of nitric oxide of plant origin. Similar to other sequenced phytopathogenic pseudomonads associated with woody plant species, the *Psa* strains isolated from leaves also display a set of genes involved in the catabolism of plant-derived aromatic compounds.

## Introduction

*Pseudomonas syringae* is a worldwide phytopathogenic microorganism mainly adapted to plant species, both monocotyledon and dicotyledon, and either cultivated or grown in wild habitats. In addition to its well-known dispersal and colonization of cultivated crops by avenues such as seeds, bulbs, bud grafting, rain and wind, there is also evidences that strains of *P. syringae* strains can be disseminated in various environments through the water cycle [1], [2] and aphids [3]. *P. syringae* strains have also been isolated from Antarctic areas [4].

The most common symptoms of *P. syringae* include leaf spots and necrosis, fruit specks and scabs, flower wilting, twig die-back, branch and trunk cankers and, in particular circumstances, plant death [5]. On the basis of visually assessed symptoms and host range tests and with the aid of biochemical, physiological and nutritional tests and molecular typing, *P. syringae* (*i.e*. the *P. syringae* species complex) is divided into 57 pathovars [6]. To genetically circumscribe 48 *P. syringae* pathovars and some related species of phytopathogenic pseudomonads, Gardan et al., [7] performed DNA-DNA hybridisation and ribotyping analyses and pointed out nine discrete genomospecies. In this study *P. s*. pv. *actinidiae* (*Psa*) was not included. By performing repetitive-sequence PCR, ARDRA and AFLP analyses, this pathovar was subsequently placed into genomospecies 8 together with *P. avellanae* and *P. s*. pv. *theae* [8], [9].

**Figure 1:**
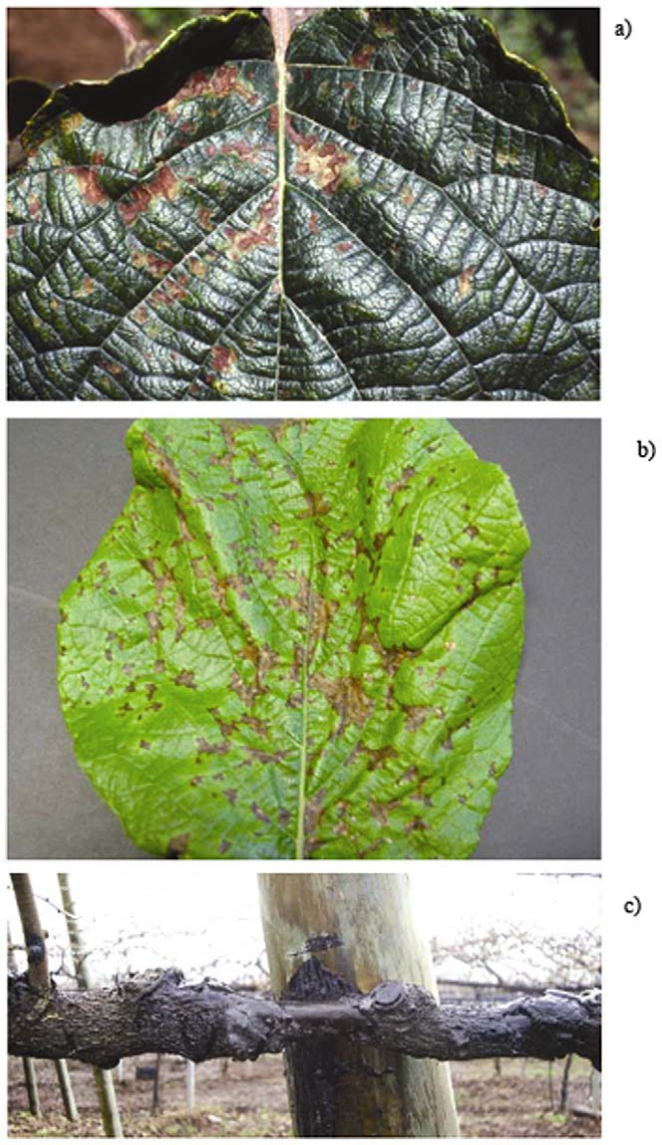
Disease symptoms of *Psa* on *Actinidia* spp. leaves and main leader. The sequenced I-*Psa* and I2-*Psa* strains from Italy were isolated from the leaves herein showed. A) Leaf symptoms on *Actinidia deliciosa* cv. Hayward (June, 1992); b) leaf symptom on *A. chinensis* cv. Hort16A (June, 2008). Note the red-rusty colour of the spots and the chlorotic halo on *A. deliciosa* and the brownish spot without halo on *A. chinensis*; c) large canker in deep winter, induced by *Psa* on the main leader of an adult *A. chinensis* cv. Hort16A plant, in central Italy (February, 2009). Note the complete destruction of all the external woody tissues of the plant.

*Psa* is the causal agent of bacterial canker of kiwigreen (*Actinidia deliciosa*), and was first reported in Japan [10]. It was then subsequently isolated in South Korea [11] and Italy [12]. In the Asian countries the pathogen caused relevant economic losses [13], whereas in Italy it has incited occasional leaf spot, twig die-back and bark canker over the past 15 years but never destructive outbreaks [14]. A bacterial canker outbreak on *A. deliciosa* in central China (Shaanxi province) was observed during 1990–1991 and reported ten years later [15]. Subsequently, another record of this disease on *A. deliciosa* was reported also in the Anhui province (Southeast China) [16]. Recently, the pathogen has been found in Portugal [17] and Chile [18]. During 2008–2011, *Psa* suddenly and very rapidly incited severe epidemics of bacterial canker in central Italy. During these epidemics the kiwigold (*A. chinensis*) was first affected and, afterwards, *A. deliciosa* [19]. *Psa* was first isolated from *A. chinensis* in southwest China (Sichuan province) in 1989 [20] and later on in southeast China (Anhui province) [21] and South Korea [22]. *Psa* was also isolated from wild *A. arguta* and *A. kolomikta* plants grown in Japan [23], [24]. In 2010, the pathogen was also found both on *A. chinensis* and *A. deliciosa* in northern Italy [25] and in France [26]. *Psa* strains identical to those found in Italy and retained very virulent to both *Actinidia* species have also been recently identified in New Zealand [27]. In Italy, the kiwigold cultivars (Hort16A, JinTao, Soreli) appear to be very susceptible and as a result, thousands of trees have dead. The main symptom of the disease are leaf spots and necrosis, extensive twig die-back, reddening of the lenticels, bleeding cankers on the trunk and leader with whitish to orange ooze (Figure 1). In Italy molecular typing, which has been performed with repetitive-sequence PCR and MLST, has revealed that there are currently clonal outbreaks of bacterial canker to both *A. chinensis* and *A. deliciosa* irrespective of the geographical areas of origin of the isolates and that the strains of the present epidemics are distinct from those causing bacterial canker on *A. deliciosa* in the past [19], [25]. As a virulence factor, some *Psa* strains produce phaseolotoxin [28], [29], which is encoded by a mobile gene cluster representing one of the first examples of horizontal gene transfer among phytopathogenic bacteria [30], [31].

These re-emerging, sudden and destructive worldwide cases of bacterial canker on highly-prized crops such as kiwigreen and kiwigold prompted us to an in-depth investigation of the genomic structure of *Psa*. Comparative genomics can provide insights into the host-pathogen interaction pathways, differential virulence factors and the chronological evolution of pathogens [32]. In recent years, complete or draft genome analyses have been performed for important phytopathogenic pseudomonads such as *P. s*. pv. *tomato* [33], [34], *P. s*. pv. *phaseolicola* [35], *P. s*. pv. *syringae* [36], *P. s.* pv. *oryzae* [37], *P. s*. pv. *tabaci* [38], *P. s*. pv. *aesculi* [39], *P. savastanoi* pv. *savastanoi* [40] and *P. savastanoi* pv. *glycinea* [41]. Sequencing multiple strains of a species or pathovar can provide important information on the possible differential evolution and adaptative mechanisms of phytopathogenic bacteria towards their hosts [34], [39], [41].

For the sequencing, we selected three representative *Psa* strains: NCPPB3739 ( =  KW 11), the type-strain of the pathovar, which was isolated in 1984 in Japan from *A. deliciosa,* cultivar Hayward [10]; NCPPB3871, which was isolated in 1992 in Italy from *A. deliciosa,* cultivar Hayward [12]; and CRA-FRU 8.43, which was isolated in 2008 in Italy from *A. chinensis,* cultivar Hort16A [19], [42]. All strains were isolated from leaf spot symptoms. These strains, which were isolated from the same organ and represent the initial outbreaks of bacterial canker on *A. deliciosa* in Japan (1984) and Italy (1992) as well as the current severe epidemics on *A. chinensis* in Italy, are good candidates for elucidating the host-pathogen relationships and the evolutionary adaptation of *Psa* towards two *Actinidia* species. The aim of this study was to investigate the biology and evolution of *Psa* strains that cause bacterial canker to different *Actinidia* species in many areas of world. We achieved this aim by performing a comparison of the genes found in the draft genomes of the *Psa* strains with other *P. syringae* pathovars and by determining the genomic variation among the *Psa* strains. We demonstrate that *Psa* shows relevant intrapathovar variations which are probably due to the gain and loss of variable genomic regions. Similar to the other sequenced phytopathogenic pseudomonads associated with woody plant species, the *Psa* strains isolated from *Actinidia* spp. leaves also display a set of genes involved in the catabolism of plant-derived aromatic compounds.

## Results

### Genome-wide sequence data

We generated genome-wide Illumina IIx sequence data from one strain of *Psa* isolated in Japan, NCPPB3739 ( =  KW 11) which is the type-strain of the pathovar and here referred to as J-*Psa*, and two *Psa* strains from Italy, NCPPB3871 and CRA-FRU 8.43, which were isolated during two different outbreaks of bacterial canker on *Actinidia* species and here referred to as I-*Psa* and I2-*Psa,* respectively. The Illumina sequencing provided nearly 10 millions of 100 nts reads that passed the quality checking. Sequencing of the J-*Psa* library provided 1,672,966 reads which were assembled into 833 contigs (N50 = 14,838; largest contig: 67,329) for a total of 5,931,199 nts (a coverage of 27.7 x). Sequencing of the I-*Psa* library provided 4,083,706 reads which were assembled into 466 contigs (N50 = 27,730; largest contig: 122,209) for a total of 5,938,909 nts (a coverage 67.6 x). Finally, sequencing of the I2-*Psa* library resulted in 3,823,264 reads which were assembled into 590 contigs (N50 = 22,372; largest contig: 85,982) for a total of 6,144,044 nts (a coverage 61.2 x). Based on the previous sequenced genomes of *P. syringae* pathovars, the obtained 6 Mb genome of *Psa* was of the expected size. The G + C content of the three strains ranges from 58.5 and 58.8% (Table 1). The sequences of the assemblies have been deposited in DDBJ/EMBL/GenBank under the following accessions: AFTF00000000 (I-*Psa*), AFTG00000000 (I2-*Psa*), AFTH00000000 (J-*Psa*).

**Table 1:** General features for *Pseudomonas syringae* pv. *actinidiae* draft genomes.

	J*-Psa*	I*-Psa*	I2*-Psa*
No. reads	1,672,966	4,083,706	3,823,264
No. contigs	833	466	590
N50	14,838	27,730	22,372
Largest contig size	67,329	122,209	85,982
Total size (bp)	5,931,199	5,938,909	6,144,044
G+C content (%)	58.8	58.8	58.5
Calculated genome coverage	27.7	67.6	61.2
Genome similarity with *Pto*DC3000 (%)	81,99	82,03	79,48

### Pairwise alignment between the draft genomes of *Psa* and the complete genome of *Pto* DC3000 and occurrence of variable regions

**Figure 2:**
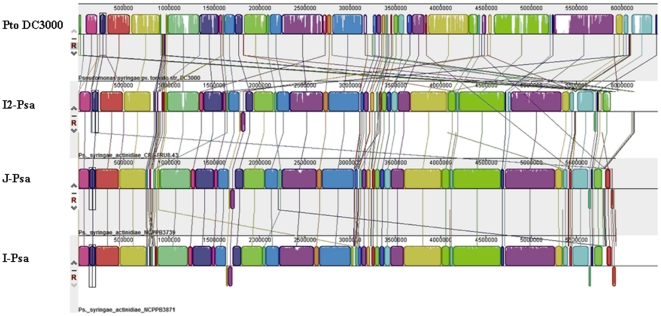
Pairwise alignment between the draft genomes of J-*Psa*, I-*Psa* and I2-*Psa* and the complete genome of *P.* *s*. pv. *tomato*DC3000 using the MAUVE software. Colored blocks outline genome sequence that align to part of another genome, and is presumably homologous and internally free from genomic rearrangement (Locally Colinear Blocks or LCBs). Areas that are completely white were not aligned and probably contain sequence elements specific to a particular genome. Blocks below the centre line indicate regions that align in the reverse complement (inverse) orientation. A profile is drawn within each LCB with the height of the color corresponding to the average degree of sequence conservation.

**Figure 3:**
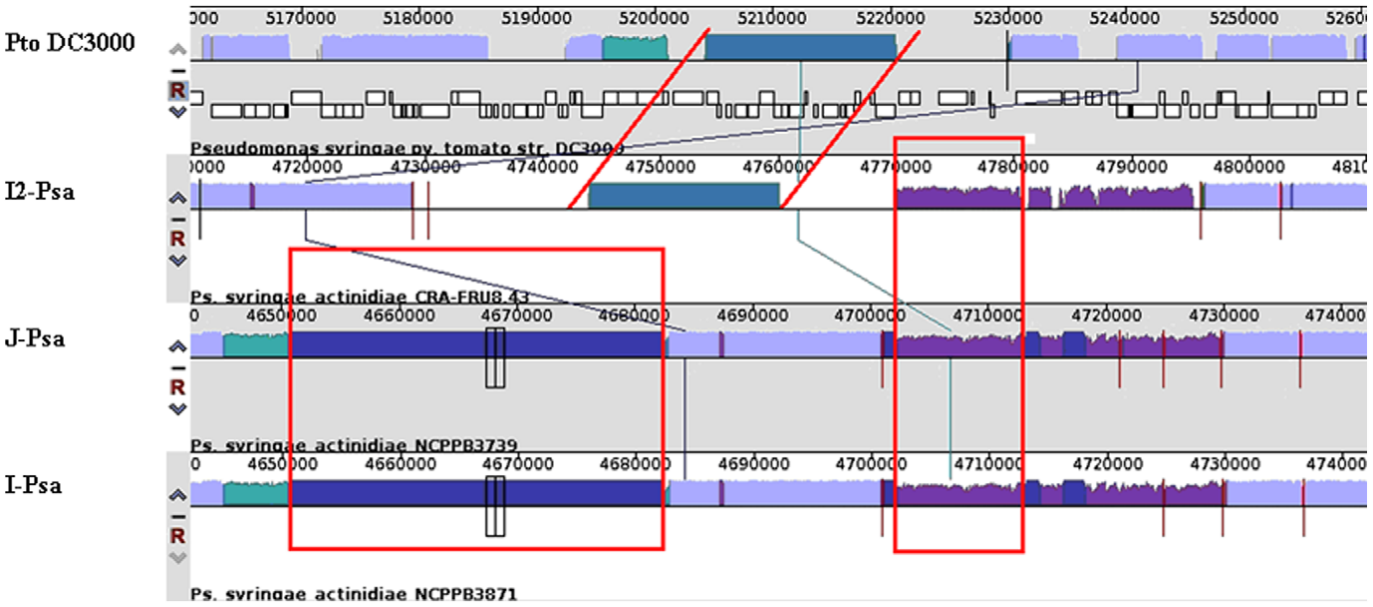
Representative part of the genome alignment between *Psa* strains and *Pto* DC3000 showing some variable regions. The violet segments (on the right) point out the variable region 3, present in all three *Psa* strains but not in *Pto* DC3000; the deep blue (on the left) segments point out the variable region 2, present in J-*Psa* and I-*Psa* but absent in I2-*Psa* and *Pto* DC3000. The blue segments indicate another variable region present in *Pto* DC3000 and I2-*Psa* but not in J-*Psa* and I2-*Psa.* The figure shows also some others shorter regions (i.e. light green segments) probable examples of horizontal gene transfer.

To investigate differences between the genomes of the three *Psa* strains and *Pto* DC3000, the closest pathovar of genomospecies 8 according to Gardan et al. [7], the draft genomes were aligned and compared using MAUVE 2.3.1. software (Figure 2). The percent similarities between the three *Psa* and *Pto* DC3000 are of 81.99, 82.03 and 79.48 for J-*Psa*, I-*Psa* and I2-*Psa*, respectively (Table 1). The alignment of the three *Psa* draft genomes with the complete genome of *Pto* DC3000 is shown in Figure 2. An *ad hoc* PERL script was used to establish the similarity of the three *Psa* genomes. The genomes of J-*Psa* and I-*Psa* resulted 99.75% and display only about 14,000 nt of differences, whereas the I2-*Psa* genome displays a similarity of 88.20% with those of the other two *Psa* strains. For each *Psa* strain, the presence of variable regions along the genomes, which are good candidates for horizontal gene transfer, were identified as regions larger than 10 kb in a contig and appeared as a gap in the genome alignment or as regions characterised by a different G+C content with respect to the average content of the three *Psa* strains. The variable regions found by comparison of the three *Psa* strains among themselves and with *Pto* DC3000 are shown in Tables 2, 3 and S1. The highest content of variable regions, namely 12, was found when I2-*Psa* was compared with J-*Psa* and I-*Psa* (Table 4). Variable region 7 was characterised by the presence of a prophage PSPPHO6, which has also been previously found *Pph* 1448A. Other evidence for the presence of mobile genetic elements has been found in variable region 8 (plasmid-partitioning protein and transposase) and 11 (phage and prophage). Variable region 2 of J-*Psa* and I-*Psa* includes the phaseolotoxin cluster (Table 3). An example of the occurrence of variable regions in the *Psa* genomes is shown in Figure 3.

**Table 2:** Variable regions (VR) found in the draft genomes of J-*Psa* (NCPPB 3739), I-*Psa* (NCPPB 3871) and I2-*Psa* (CRA-FRU 8.43) compared with the complete genome of *Pto* DC3000.

VR	Strains	Contigs	Coordinate	% GC	VR-encoded genes
1	CRA-FRU8.43A	Contig234	from start to 11429	56.1	Glycosyl transferase, group 1 family protein
	NCPPB3739	Contig729	from start to 11429	56.2	HlyD family secretion protein
	NCPPB3871	Contig248	from 18 to 11447	56.2	Mannose-1-phosphate guanylyltransferase/mannose-6-phosphate isomerase
					Outer membrane efflux protein
					Type I secretion system ATPase, PrtD
2	CRA-FRU8.43A	Contig65	from 16690 to 32252	62	11 putative type III secretion system component
	NCPPB3739	Contig226	from 17561 to 33132	62	3 hypothetical protein
	NCPPB3871	Contig60	from 80641 to 96212	62	LuxR family transcriptional regulator
					Myosin heavy chain B (MHC B)
					No database match
					TPR domain-containing protein
					Type III secretion system protein
3	CRA-FRU8.43A	Contig50	from 15151 to 25647	57.3	2 Prepilin
	NCPPB3739	Contig515	from 1455 to 11968	57.2	2 Type II secretion system protein E
	NCPPB3871	Contig262	from 11911 to 22424	57.2	No database match
					Type II and III secretion system protein
					Type II secretion system protein
					Type IV pilus protein
4	CRA-FRU8.43A	Contig130	from 150 to 13257	57.9	2 No database match
	NCPPB3739	Contig771	from 194 to 13301	57.9	Filamentous hemagglutinin
	NCPPB3871	Contig401	from 195 to 13302	57.9	Hemolysin activator protein precursor
5	CRA-FRU8.43A	Contig175	from start to 15590	60	ABC transporter, periplasmic oligopeptide-binding protein
	NCPPB3739	Contig46	from start to 15590	60	ABC transporter, permease protein
	NCPPB3871	Contig35	from start to 15590	60	Achromobactin biosynthetic protein AcsD
					Conserved hypothetical protein
					Diaminobutyrate--2-oxoglutarate aminotransferase
					Dipeptide ABC transporter, ATP binding protein
					Dipeptide transporter dppD-like protein
					Hypothetical protein RL0789
					No database match; (similar to of nd with eval = nd)
					PupR protein
					Putative transporter, permease protein
					RNA polymerase, sigma-24 subunit, ECF subfamily
					Sigma-70 region 2
					TonB-dependent siderophore receptor
6	CRA-FRU8.43A	Contig175	from 15597 to 27012	62.1	Achromobactin biosynthetic protein AcsB
	NCPPB3739	Contig96	from start to 11415	61.8	Achromobactin biosynthetic protein AcsC
	NCPPB3871	Contig35	from 15592 to 27007	61.9	Achromobactin-binding periplasmic protein precursor
					Achromobactin transport ATP-binding protein CbrD
					Achromobactin transport system permease protein CbrB
					Achromobactin transport system permease protein CbrC
					Dimethylmenaquinone methyltransferase
					Drug resistance transporter EmrB/QacA subfamily
					Hypothetical protein
					Orn/DAP/Arg decarboxylase 2:Orn/DAP/Arg decarboxylase 2
					LucA/IucC

**Table 3:** Variable regions (VR) found in the draft genomes of J-*Psa* (NCPPB 3739) and I-*Psa* (NCPPB 3871) compared with I2-*Psa* (CRA FRU 8.43) draft genome.

VR	Strains	Contigs	Coordinate	% GC	VR-encoded genes
1	NCPPB3739	Contig 367	from 3002 to 14705	59.5	2 No database match
	NCPPB3871	Contig 71	from start to 11703	59.5	Dak phosphatase
					Glycerone kinase
					Iron-sulfur cluster-binding protein, Rieske family
					Periplasmic binding protein/LacI transcriptional regulator
					Putative sugar-binding region
					Quinoprotein
					Ribose ABC transporter, ATP-binding protein
					Ribose/galactose isomerase
					Short chain dehydrogenase
					Sorbitol dehydrogenase, putative
					Sugar ABC transporter, ATP-binding protein
2	NCPPB3739	Contig 717	from 11215 to 49219	52.4	13 Hypothetical protein
	NCPPB3871	Contig 151	from 18056 to 56060	52.4	2 Deoxycytidine triphosphate deaminase
					2 Fatty acid desaturase
					2 ISPsy25, transposase
					2 Phage integrase family site specific recombinase
					HAD superfamily hydrolase
					L-arginine:lysine amidinotransferase, putative
					Ornithine aminotransferase
					Phaseolotoxin-insensitive ornithine carbamoyltransferase
					pyruvate phosphate dikinase PEP/pyruvate binding subunit
					RtrR protein
3	NCPPB3739	Contig 248	from 1965 to 37060	57.8	13 Hypothetical protein
	NCPPB3871	Contig 169	from 1965 to end	57.8	3 Conserved hypothetical protein
					3 No database match
					Bacteriophage-related protein
					Baseplate assembly protein J
					Baseplate assembly protein W
					Baseplate assembly protein V
					Bifunctional DNA primase/polymerase
					Deoxynucleotide monophosphate kinase
					Holin
					Lysozyme
					Major capsid protein E
					Major tail sheath protein
					Major tail tube protein
					P2-like prophage tail protein X
					Phage DNA packaging protein, Nu1 subunit of terminase
					Phage late control gene D protein
					Phage protein U
					Portal protein, lambda family
					Prophage PSPPH06, adenine modification methytransferase
					Prophage PSPPH06, site-specific recombinase, phage integrase family
					Prophage PSPPH01, transcriptional regulator
					Tail fiber protein H, putative
					Tail protein I
					Tail tape meausure protein
					Terminase, large subunit
					Transcriptional regulator
					Transcriptional regulator, TraR/DksA family

**Table 4:** Single Nucleotide Polymorphisms (SNP) found among the three *Psa* strains, *P. avellanae* BPIC631 and *P. s.* pv. *theae* NCPPB2598 draft genomes in the genes that were found polymorphic between I-*Psa* and J-*Psa*. ORF names refer to the I-*Psa* genome draft.

ORF	Position in ORF	I-*Psa*	J-*Psa*	I2-*Psa*	*Psth*	*Pav*
orf00015-contig244	761	T	G	T	T	T
orf00040-contig34	3138	T	G	T	T	T
orf00010-contig65	51	G	C	G	G	G
orf00018-contig32	24	C	A	A	C	§
	30	A	G	G	A	§
	39	T	C	C	C	§
	543	G	T	G	G	§
orf00020-contig429	639	C	G	C	C	C
	642	C	G	C	C	C
orf00001-contig216	1628	C	T	C	C	C
	1632	A	G	A	A	A
	1670	T	G	G	G	G
	1674	A	G	G	A	A
	1824	T	C	C	T	T
	1829	A	G	A	A	A
	1830	A	G	G	A	A
orf00007-contig330	92	T	G	T	G	G

Ancestral residues (identified as the residues displayed by *P. avellanae* and *P. s.* pv. *theae*) are highlighted. *Pav*  =  *P. avellanae; Psth*  =  *P. s.* pv. *theae. § = * orthologous not found.

### *Psa* is phylogenetically closer to *P. s*. pv. *theae* than to *P. avellanae*

**Figure 4:**
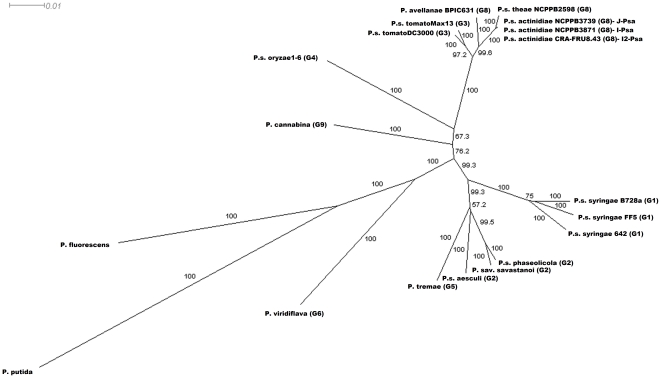
Evolutionary relationships of *Psa* strains to other phytopathogenic pseudomonads. Phylogenetic relationships were estimated from concatenated sequences from three housekeeping genes, *gyrB*, *rpoB* and *rpoD* (1,646 bp), using the neighbour-joining (NJ) algorithm. Bootstrap values are reported at each branching. Members of all nine genomospecies (Gardan et al., 1999), except genomospecies 7, are included into the analysis. The letter followed by the number reported in brackets indicates the genomospecies *sensu* Gardan et al. [7].

To establish phylogenetic relationships between *Psa* and the other pathovars or species of the *P. syringae* complex, we used MultiLocus Sequence Type (MLST) analysis. The relationships within the nine genomospecies, which were determined by Gardan et al. [7], were assessed by considering as many strains of each genomospecies for which the complete or partial sequence of orthologous housekeeping genes was deposited in the NCBI databank as possible. For members of eight out of nine genomospecies, we constructed a concatenated dendrogram based on the neighbour-joining (NJ) algorithm using the *gyrB*, *rpoB* and *rpoD* gene fragments for a total of 1,646 nucleotides. For genomospecies 7 (i.e. *P. s*. pv. *tagetis* and *P. s*. pv. *helianthi*), there were not enough gene sequences in the databank, and consequently, it was not included in the analysis. The phylogenetic tree is shown in Figure 4. *Psa* appears closely related to *P. s.* pv. *theae*-type strain and slightly distant from *P. avellanae*-type strain, which are the other two members of genomospecies 8. Genomospecies 3 (*P. s*. pv. *tomato*) is the most related to the genomospecies 8, as has already established by Gardan et al., [7]. Furthermore, we analysed the phylogenetic position of the three *Psa* strains among the *P. syringae* pathovars using more orthologous genes. In addition, a concatenated tree, based on the maximum likelihood (ML) algorithm and the *acnB, fruK, gltA, pgi, rpoB* and *rpoD* gene fragments for a total of 2,926 nucleotides, was also built (Figure S1). Again, the *Psa* strains are more closely related to *P. s*. pv. *theae* than to *P. avellanae*. In addition, I2*-Psa* appears to not be identical to the other two *Psa* strains sequenced here. The high genetic similarity between *Psa* and *P. s*. pv. *theae* observed in the present and previous studies, the records of bacterial canker incited by *Psa* from both cultivated and wild *Actinidia* species in eastern Asia countries [10], [11], [13], [15], [16], [20]–[24] and the fact that *P. s*. pv. *theae* has, so far, been solely reported solely in Japan, led us to postulate that these two closely-related *P. syringae* pathovars also have their origin in eastern Asia, as has already been hypothesised by Ushiyama et al. [23]. The consideration that the *Actinidiaceae* and *Theaceae* families, which are genetically closely related, both originated in eastern Asia would support such an hypothesis.

### *Psa* harbors putative pathovar-specific mobile genetic elements of potential importance in adaptation to *Actinidia* spp

With the total protein complement of the *Psa* strains, we focused on putative proteins encoded by the genome of each strain that showed no significant homologies with proteins encoded by previously sequenced genomes of phytopathogenic pseudomonads. In particular, all the *Psa* strains display putative phage integrases, integrase family proteins, and transposases. Interestingly, all the *Psa* strains have putative homologues to the cAMP protein Fic which induces filamentation in the bacterial cells. Such proteins have been found in the *Dickeya zeae* strain ECH1591, which causes soft rot diseases, but have never been reported in phytopathogenic pseudomonads.

### Comparison of the protein complement of *Psa*

Because figures calculated by MUMMER may be severely overestimated due to the misassemble of the short Illumina reads that may occur in correspondence to repeated sequences, we used the predicted protein complements to estimate the differences among the three *Psa* strains. An ORF search using GLIMMER predicted 5,670 genes in the J-*Psa* genome draft which included 20.37% hypothetical proteins and 3.54% conserved hypothetical proteins, 5,557 genes in I-*Psa* which included 20.54% hypothetical proteins and 3.74% conserved hypothetical proteins and 5,714 genes in I2-*Psa* which included 22.72% hypothetical proteins and 3.48% conserved hypothetical proteins. For each strain, all predicted proteins were preliminarily annotated by homology search in the RefSeq database and ordered by role categories according to TIGRFAMs. Here we show the protein categorisation of I2-*Psa* (Table S2). In agreement with the results of the genome-wide comparisons reported above, the resultant putative protein complement of I-*Psa* and J-*Psa* were highly similar. As many as 5,076 ORFs were found to be 100% identical in their DNA sequence between the two strains and only seven ORFs were found to be polymorphic, which accounted for a total of 18 SNPs (Table 4), if two cases of ambiguity due to presumptive gene duplication are excluded. The remaining predicted proteins were found to be different from the reciprocal strain due to the differential fragmentation of the draft genomes into contigs or to occasional misassemble, i.e. a small number of predicted ORFs (38 in the J-*Psa* comparison with I-*Psa* and 52 in the reciprocal comparison) were not detected in the reciprocal strain as such, but in all cases BLASTn analysis showed that the orthologous sequences were present and had likely been neglected by GLIMMER due to contig fragmentation and consequent loss of signals needed by the program for prediction. Conversely, strain I2-*Psa* showed significant differences when compared to I-*Psa* and J-*Psa.* In the comparison versus J-*Psa* 2,083 ORFs were identical while 2,140 showed sequence polymorphisms (SNPs). The remaining ORFs were further analysed and, in most cases were found to differ in length. However, whether this difference was due to actual length polymorphism or differential fragmentation of the draft genome into contigs could not be determined. For as many as 398 ORFs, however, no sequence with significant similarity could be found in the I-*Psa* or in the J-*Psa* genome draft.

### Origin and evolutionary relationships among *Psa* strains

Further analysis of these I2-*Psa* specific ORFs was performed to determine their origin. We searched the 35 *Pseudomonas* spp. and pathovar genomes available as draft or as complete genome sequences from NCBI as well as the draft genomes of *P. avellanae* BCIP613 and *P. syringae* pv. *theae* NCPPB2598, type-strains of these pathogens, whose draft genomes are available (Marcelletti, Firrao, Scortichini, unpublished data in these labs) for comparison using BLASTn. The results of this search showed that among the 398 I2-*Psa*-specific ORFs, there were 238 ORFs for which no homolog could be found in *P. avellanae* BCIP 613 or *P. s.* pv. *theae* NCPPB2598. It was also found that 49% (i.e. 196) of the 398 proteins that do not have homologous in J-*Psa* or I-*Psa* matched sequences in at least one of the genomes of *Pto* strains assessed, specifically 158, 144, 138, 114 and 116 matches for *Pto* strains K40, Max13, NCPPB1108, T1 and DC3000, respectively. These evidence as well as the annotation of several of the deduced proteins as phage or prophage proteins strongly suggests that a large part of this I2-*Psa-*specific DNA has been acquired by horizontal gene transfer from a strain of *P. syringae* genomospecies 3. However, within the group of 398 I2-*Psa* specific DNA, we also detected eight ORF that had significant BLASTn hits in the *P*. *avellanae* BCIP613 or *P. s.* pv. *theae* NCPPB2598 draft genome sequences but no hit in any of the 35 *Pseudomonas* spp. and pathovars genome sequences available from NCBI (Table 4). The most obvious explanation for this result is that these eight sequences shared by I2-*Psa* and other strains of genomospecies 8 were lost by I-*Psa* and J*-Psa* during their evolution.

To gain access to further information about the origin of I2-*Psa*, we analysed the DNA sequences of all the genes that were polymorphic in all three *Psa* strains and compared them with their orthologous in the *P. avellanae* BCIP613 or *P. s.* pv. *theae* NCPPB2598 draft genome sequences. As reported in the Table 4, although the pattern displayed by I2-*Psa* was more similar to I-*Psa* than to J-*Psa*, in most cases I2-*Psa* displayed the ancestral residue in common with the other strains of genomospecies 8.

**Figure 5:**
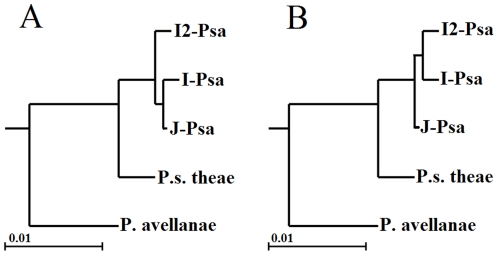
Genealogy of strains of genomospecies 8. (A) Maximum likelihood tree resulting from the analysis of a concatenation of 171 ORFs. (B) An alternative genealogical hypothesis with the constrain of the common origin of the Italian *Psa* strains.

Furthermore, we randomly selected 171 ORFs from the list of genes that were found to be orthologues among the three strains and in the draft genomes of *P. avellanae* BCIP613 or *P. s*. pv. *theae* NCPPB2598. The alignment of the concatenation of these genes, which consisted of 166,160 nucleotide positions was used to determine the genealogy with maximum likelihood and perform hypothesis testing. Based on the results, as shown in the tree in Figure 5a, the I2-*Psa* strains originated from a common ancestor of the I-*Psa* and J-*Psa* strains and was not a derivative of either of these strains. To provide statistical support to this presumptive genealogy, we estimated the likelihood of an alternative genealogy with the constraint that I2-*Psa* and I-*Psa* were monophyletic (Figure 5b), and performed a test for monophyly to evaluate whether such a null hypothesis could be rejected with statistical significance. The aim of the monophyly test is the evaluation, by means of parametric bootstrap, of the significance of a likelihood ratio calculated comparing a null hypothesis with the unconstrained maximum likelihood tree. The log likelihoods of the null hypothesis and the unconstrained tree were -263874.12 and -263011.44, respectively, and their ratio (Δ = 1725.36) was compared with the delta distribution in a set of alignments of simulated sequences evolved *in silico* using the unconstrained tree as guidance. The comparison showed that the null hypothesis was to be rejected (P = 0.016). In summary, the analysis of ancestral state residue conservation, the maximum likelihood analysis of genealogies and the evidence of eight ORFs that are genomospecies 8-specific and are present only in I2-*Psa* strongly suggest that these strains did not evolve from the organisms that caused the epidemic in 1984–1992 but rather from a common ancestor.

### Secretion systems associated with pathogenicity and virulence

The three *Psa* strains sequenced display structural genes involved in the biosynthesis of the type I, II, III, IV and VI secretion systems (TSS). The T1SS encodes for orthologues of the HlyD family secretion protein involved in the transportation of the *Escherichia coli* α-haemolysin toxin. Both the general secretion Sec-pathway and the Tat-pathway of the T2SS are present in the three *Psa* strains. These two secretion systems are also present in the other sequenced plant pathogenic pseudomonads, even though the T1SS is not present in *Pto* DC3000 [42]. In *Pto* DC3000, the twin-arginine translocation (Tat) system of the T2SS appears to be an important virulence determinant [43]. A complete T3SS, similar to those found in the *P. syringae* complex [44] has also been found in the three *Psa* strains. The T3SS contains the *hrp/hrc* cluster and the transcriptional regulatory HrpL, HrpR and HrpS proteins. TrbC and VirK protein orthologues for the T4SS are present in the three *Psa* strains. In addition, I2*-Psa* also displays other orthologous proteins for the T4SS, namely TraG and VirB8, which are also present in other *P. syringae* pathovars. Noteworthy the GC contents of these genes are lower than the flanking genomic regions, this indicating the occurrence of possible genomic islands. Finally, the three *Psa* strains also display two clusters of orthologous proteins of the T6SS: the Vgr family protein, which is identical to that of *P. putida* GB-1 and the OmpA family protein which is required for the T6SS functionality [45]. ImpA, an inner membrane protein of the T6SS is also present. The precise functions of these secretion systems in *Psa* have yet to be investigated.

### Type III secretion system effectors

**Figure 6:**
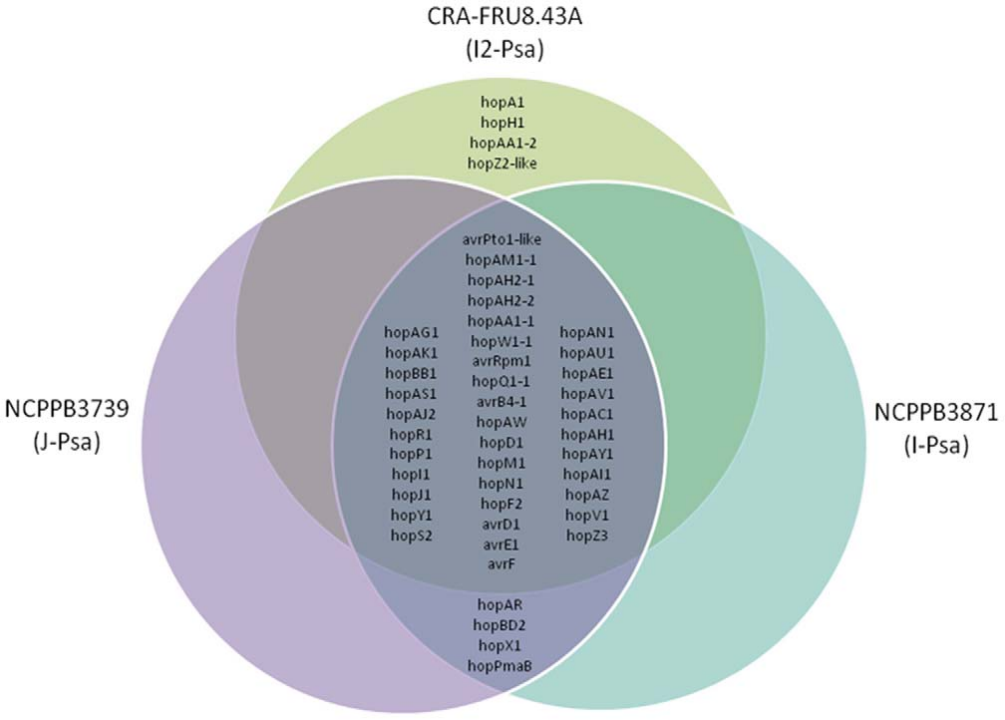
Venn diagram of the type III effector gene complements of J-*Psa*, I-*Psa* and I2-*Psa* strains based on the comparison of the same complement of other sequenced plant pathogenic pseudomonads. The genes conserved among the three strains are indicated in the middle of the diagram. J-Psa and I-Psa as well as I2-Psa display four unique different effector genes (see also Table S3).

A comparison of the effector repertoire of the three *Psa* strains based on the complete effector repertoire of *P. s*. pv. *tomato* DC3000, other *P. syringae* strains and *Psa* MAFF302091 reveals a “core” set of 33 *hop* and 6 *avr* putative effector genes that are conserved in all strains (Figure 6). In general, in these putative effectors, the amino acid identity is very high (i.e. >90%) to the most similar orthologue of the *P. syringae* pathovars found in GenBank (Table S3). However, for *hopAC1*, *hopAE1*, *hopI1*, *hopM1*, and *hopZ3,* the amino acid identity with respect to their most similar orthologues ranged from 68 to 79%. By contrast, the *avrPto1-*like effector of the three *Psa* strains shows an amino acid identity of 44% with the orthologue of *P. s*. pv. *aesculi* NCPPB3681. All three *Psa* strains did not show the presence of *hopAB* and *hopAF* which are considered conserved effector genes present in *Pto* DC3000, *Pph* 1448A and *Psy* B728a [46]. Interestingly, I*-Psa* displays four putative effector genes, namely *hopA1, hopAA1-2, hopH1*, and *hopZ2-*like, that are not present in the J-*Psa* and I-*Psa* strains. The first three putative effector genes display a relative similarity with the orthologues of *Pto* DC3000 (91%), *Pto* DC3000 (99%) and *P. s*. pv. *tomato* T1 (97%), whereas *hopZ2*-like shows a similarity of 40% with the orthologue of *P. s.* pv. *syringae*. *HopA1* triggers effector-triggered immunity in tobacco, other *Nicotiana* species [47] and in many *Arabidopsis* accessions [48] and is supposedly involved in host range specificity, even though its virulence functions are still unknown [49]. *HopAA1-2* is a paralogue of the *hopAA1-1* effector, which is located in the conserved effector locus (CEL) region of the *hrp/hrc* cluster and is considered to be among the ancient *P. syringae* effectors that were acquired before the radiation of *P. syringae* into the current pathovars [50]. *HopAA1-2* is present in *Pto* DC3000 but not in *Pto* T1, *Pph* 1448A and *Psy* B728a. Evidence for a strong virulence function for *hopAA1-2* in plants has yet to be fully investigated [51]. Notably, in a wide assessment on the presence/absence of effector genes in 91 strains of *P. syringae* pathovars, Sarkar et al. [52] found that *hopAA1-2* and *hopA1* are among the least distributed effector genes. In fact, these effectors have only been found in eight and 16 out of the 91 strains tested, respectively, and they have only been found present together in three *Pto* strains. If these two effectors can explain the relevant aggressiveness of I2*-Psa* towards *A. chinensis* and *A. deliciosa* they deserve further in-depth studies. *HopH1* is considered to be a variably distributed effector gene in the *P. syringae* pathovars [53]. The *hopZ2*-like effector protein showed a low similarity (i.e. 44%) with *hopZ2*. The possibility that this putative protein represents a different and new effector gene cannot be ruled out. Finally, I2-*Psa* lacks *hopX1*, another conserved effector present in other *P. syringae* pathovars [46] as well as in J*-Psa* and I-*Psa*. These results would indicate that both *Actinidia deliciosa* and *A. chinensis* can be infected by different *Psa* strains displaying different arrays of effector genes and represents a case of convergent evolution of closely-related pseudomonads to the same host genus.

### Variation in plasmid content among *Psa* strains

**Figure 7:**
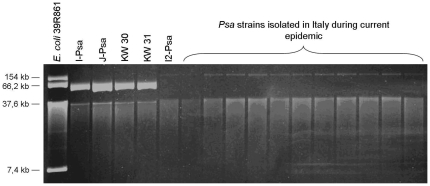
Plasmid profiles of *Psa*. Agarose gel electrophoresis to compare the number and size of native plasmids in the genome of *Psa* strains. The gels show also other representative *Psa* strains from the outbreak of bacterial canker in Japan (i.e. 1984) and from the current severe epidemics in Italy. See also Materials and Methods. Note as the ca. 50 kb plasmid present in J-*Psa* and I-*Psa* is not contained in all the *Psa* strains isolated from the current epidemic in Italy. By contrast, I2-Psa and other strains obtained from the recent epidemics of bacterial canker in Italy contain a plasmid of about 160 kb.

Using agarose gel electrophoresis, we compared the number and the size of native plasmids present in the three sequenced *Psa* strains as well as for comparative purposes, in other representative *Psa* strains from Japan (i.e. outbreaks of 1984) and Italy (i.e. outbreaks of 2008–2010 in different Italian regions). We found that all the Japanese strains from the 1984 outbreak as well as I-*Psa* harbour a native plasmid of about 50 kb. This plasmid is absent in I2-*Psa* and in all the other representative *Psa* strains isolated in Italy during the recent epidemics of bacterial canker on *A. chinensis* and *A. deliciosa* (Figure 7). Remarkably, I2-*Psa* as well as all the other *Psa* strains obtained during the recent epidemics of bacterial canker in Italy displayed the presence of a plasmid of about 160 kb, not present in both J-*Psa* and I-*Psa*. This represent a substantial acquisition of genetic material for I2*-Psa*. All the three *Psa* strains has the the *repA* gene, essential for plasmid replication, of the *P. syringae* pPT23A-like plasmid family.

### Presence and absence of the phaseolotoxin gene cluster and other toxins

The phaseolotoxin gene cluster, *argK-tox*, is located on the chromosome of *Pph* and *Psa.* It comprises three tyrosinase-recombinase-encoding genes: *txi1, txi2* and *txi3*, which are located at the left end of the cluster; the *phtE* locus, which contains ORFs showing homologies to genes encoding amino acid transferases and ARAC family and fatty acid desaturase; and the *argK* gene which encodes the phaseolotoxin-resistant ornithine carbamoxyltransferase. The cluster is flanked by two regions, ACT059 and ACT094 [54]. Thirty-eight kilobases of the *argK-tox* cluster of the *Pph* MAFF302282 and *Psa* NCPPB3739 (i.e. J-*Psa*) strains are identical [54]. In our draft, we found that J*-Psa* and I-*Psa* contain the phaseolotoxin gene cluster, which display homologies of 99.94% and 99.99%, respectively, whereas I2*-Psa* does not contain this 38 kb region of the cluster and the flanking regions, ACT059 and ACT094, are contiguous (Figure S2). Some genetic features indicate that the phaseolotoxin cluster, also referred to as the *tox*-island, has been acquired by *P. syringae* pvs. *phaseolicola* and *actinidiae* through horizontal gene transfer from an unknown species [31], [55]. The complete absence of the cluster in many highly virulent *Psa* strains, as found both in Italy and New Zealand [19], [27] would confirm such a hypothesis and indicate the remarkable aggressiveness of the pathovar even without this virulence factor. These findings also indicate the separate but convergent evolution of genetically different pseudomonads as phytopathogens of *Actinidia* spp. Notably, different types of leaf spot lesions on *Actinidia* spp. have been noticed during the two outbreaks of bacterial canker of kiwifruit in Italy in 1992 and 2008-2011 (Figure 1), even though necrotic spots surrounded by a chlorotic halo have also been frequently observed during these severe epidemics on *A. chinensis* in Italy. The presence of genes coding for coronatine, syringomycin and syringopeptin toxins was also checked but none of these toxins is present in the draft genomes of the *Psa* strains of the present study.

### Copper resistance and antibiotic detoxification

A thorough search for orthologous genes coding for resistance to copper and antibiotics was performed with the draft genomes of the three *Psa* strains. All strains contain homologues for the *copA* and *copB* genes, which are essential for copper resistance [56]. In *P. syringae, copA* is located in the periplasmic space, whereas *copB* is in the outer membrane of the bacterial cell. The other two genes, namely *copR* and *copS*, which are required for maximum copper resistance were not found. These results confirm a study by Nakajima et al. [57] who found only *copA* and *copB* in the *Psa* strains, including NCPPB3739, obtained during the initial outbreak of bacterial canker on *A. deliciosa* in Japan when the regular spraying of copper bactericides was not yet applied to the infected orchards. In addition, all the *Psa* strains display a vast set of orthologous genes involved in antibiotic resistance. Among the five superfamilies of efflux transporters, *Psa* has genes belonging to the resistance nodulation division (RND), multi antimicrobial resistance (MAR), multidrug endosomal transporter (MET) and major facilitator superfamily (MFS). Recent studies on the antibiotic resistance mechanism in Gram-negative bacteria, have stressed that resistance greatly depends on the constitutive or inducible expression of active efflux systems [58]. As an example, the disruption of *MexB*, a gene of the MAR superfamily, that is also present in *Psa* strains, dramatically increased the susceptibility of *P. aeruginosa* to beta-lactams, tetracyclines, fluoroquinolones and chloramphenicol [58]. In addition, all the *Psa* strain genomes include genes involved in the enzymatic inactivation of beta-lactams, tellurium and a macrolide ABC efflux protein, *macAB*, which confers resistance to cycloheximide. All the *Psa* strain genomes contain a*mpD*, a gene coding N-acetyl-anhydromuramyl-L-alanine amidase which cleaves the amide bond between N-acetylmuramoyl and L-amino acids in the bacterial cell wall. Finally, I2-*Psa* also contains a putative lantibiotic dehydratase domain that has never been found in other phytopathogenic pseudomonads.

### Iron acquisition, nitric oxide and sucrose metabolism and quorum sensing

The three *Psa* strain genomes encoded a number of genes involved in iron acquisition, such as the siderophore pyoverdine and enterobactin involved in the isochorismate synthase and yersiniabactin, a siderophore with a very high affinity for iron. In addition, all the genomes contain the TonB protein. This protein spans the periplasm and is anchored to the cytoplasmic membrane interacting with receptors in the outer membrane to facilitate the uptake of iron-siderophore complexes. In human bacterial pathogens, namely *Haemophilus influenzae* and *H. parainfluenzae*, the inactivation of TonB decreased the ability to cause disease [59]. The three *Psa* strains contain two genes involved in the nitric oxide metabolism, namely nitric oxide dioxygenase and anaerobic nitric oxide reductase, with 100% homology to the same genes in *P. s*. pv. *aesculi* [39]. These genes might protect *Psa* from the host defence responses incited by nitric oxide. The three *Psa* strains do not have the 8 kb cluster coding for sucrose utilization that is present in the phloem infecting *P. s*. pv. *aesculi* strain 2250 isolated in Great Britain [39]. The quorum sensing system of *Psa* apperas to differ from the classic LuxR/LuxI of other *P. syringae* strains. In fact, the genes of LuxI family are absent from these three strains, which display putative LuxR family genes. The repressor genes *rsaM* and *rsaL* are also absent in the three *Psa* genomes.

### Presence of pectolytic enzymes and catabolism of plant-derived aromatic compounds

The three *Psa* strains contain pectin lyase and polygalacturonase genes that display complete identity to the orthologues of *Pto* T1. These enzymes are also present in the soft-rot bacterium *P. marginalis* [60]. Similar to other *P. syringae* pathovars that infect woody hosts, such as *P. s*. pv. *aesculi* and *P. savastanoi* pv. *savastanoi* [39], [40], *Psa* also displays genes involved in the catabolism of plant-derived aromatic compounds using both the cathecol branch of the β-chetoadipate and the protocatechuate 3,4-dioxygenase pathways. In fact, all the *Psa* genomes encode putative proteins involved in the degradation of anthranilate to catechol (i.e. anthranilate dioxygenase reductase and anthranilate phosphoribosyltranferase) as well as proteins involved in the catabolism of cathecol (cathechol 1,2-dioxygenase, muconolactone delta-isomerase, muconate cycloisomerase N-terminal and dienelactone hydrolase. Moreover, the *Psa* strains have putative *pcaG* and *pcaH* genes encoding the two subunits (α and β) of protocatechuate 3,4-dioxygenase, an enzyme involved in the degradation of protocathecuate, which is present in soil-inhabiting bacteria.

### Differential multiplication trend of *Psa* strains in *Actinidia* spp. leaves and pathogenicity test to tomato

**Figure 8:**
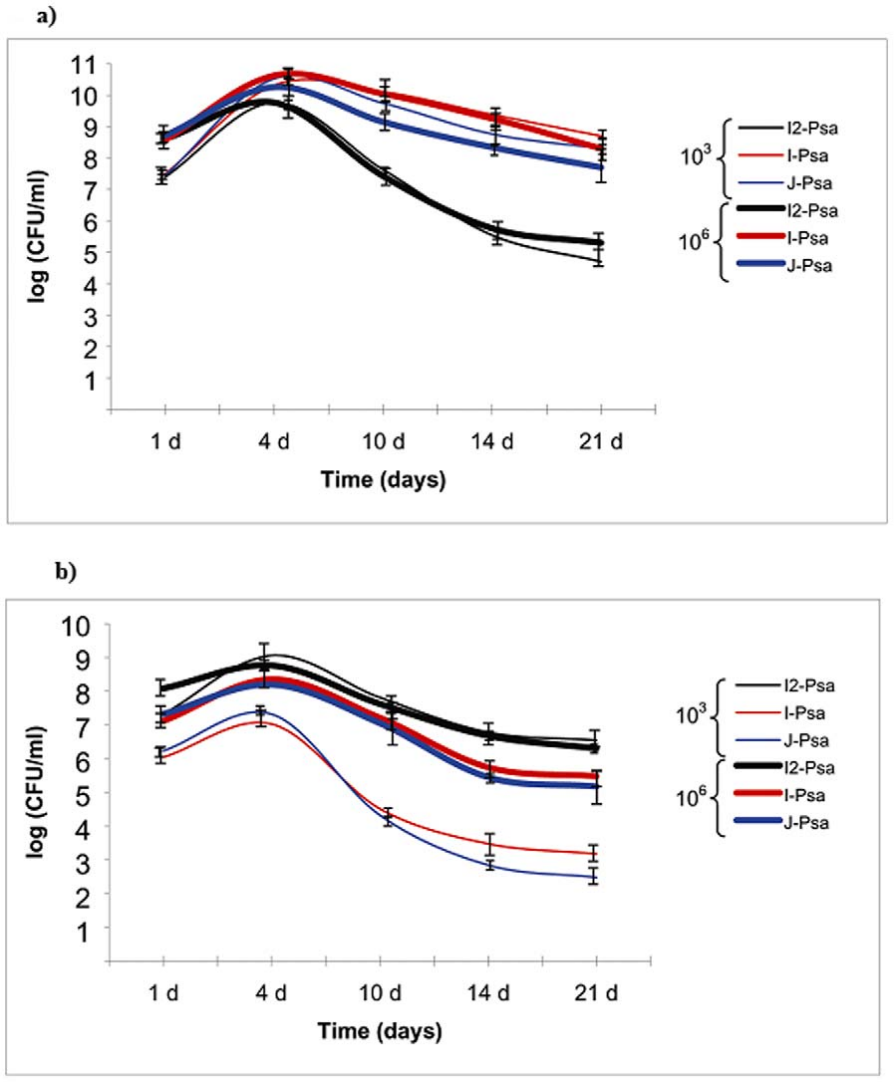
Multiplication trends of *Psa* strains in *Actinidia* species. Multiplication in *A. deliciosa* cv Hayward (a) and in *A. chinensis* cv Hort16A (b) leaves. Bacteria were inoculated at 1–2×10^3^ and 1–2×10^6^ cfu/ml. Data represent the mean log of bacterial cell number and standard deviation (SD) as obtained from eight inoculation sites per each sample.

Field evidence from the recent epidemics of bacterial canker in Italy indicate that the current *Psa* population (I2-*Psa*) is aggressive to both *A. chinensis* and *A. deliciosa.* In the latter species, I2*-Psa* incited more severe symptoms compared with the *Psa* population of the past outbreaks in Italy (I-*Psa*) when solely *A. deliciosa* was cultivated. Inoculations of *A. deliciosa* and *A. chinensis* leaves revealed different multiplication trends between J-*Psa*, I-*Psa* and I2-*Psa* (Figure 8). In fact, I2-*Psa* performed better than J-*Psa* and I-*Psa* in *A. chinensis* leaves. I2-*Psa* performed well also on *A. deliciosa*, although not as well as I-*Psa* and J-*Psa*, as three weeks after the inoculation still showed high cell levels (i.e. between 10^5^ and 10^6^ cfu/ml). Conversely J-*Psa* and I-*Psa,* that reached a cell concentration of about 10^10^ cfu/ml 3–4 days after the inoculation with either 10^3^ or 10^6^ cfu/ml when inoculated in *A. deliciosa* leaves, only grew poorly in *A. chinensis* leaves where the cell concentration of the strains decreased significantly 21 days after the inoculation. The control leaves did not show any sign of infection. These results indicate that the *Psa* population currently causing severe damage in Italy and New Zealand is capable to infect both *Actinidia* species, whereas the *Psa* population causing outbreaks of bacterial canker to cv. Hayward in Japan and Italy about 20–25 years ago displays more affinity for *A. deliciosa*. The three *Psa* strains incited a typical hypersensitivity reaction on tomato leaves, whereas *Pto*DC3000 caused typical symptoms of bacterial speck.

## Discussion

Through Illumina sequencing technology, we have performed a genome-wide survey of genetic variation in *Psa* strains, the causative agent of bacterial canker of *Actinidia* spp. worldwide. This study has determined putative variable genomic regions and sets of genes related to the pathogenicity and virulence that could differentially modulate the aggressiveness of pathogen populations towards *Actinidia* species as well as genes involved in the environmental fitness and adaptation of the bacterium *in planta*. We confirmed that the re-emerging wave of bacterial canker to *A. deliciosa* and *A. chinensis* is being raised by a population of *Psa* (i.e., I2-*Psa*) distinct from the population that has led to past outbreaks in Japan and Italy (i.e. J-*Psa* and I-*Psa*) [10], [12]. Moreover, we stress that the current epidemics of bacterial canker are being caused by a *Psa* population that, most probably, did not originate from that found in Italy about 20 years ago. The origin of this new epidemic wave has rised a large debate worldwide, and it has not been easy to provide convincing answers using conventional approaches. Conversely, the large amount of sequence data obtained in this work has provided unmatched solidity to the reconstructed genealogy of the *Psa* strains examined.

We refined the taxonomic position of *Psa* within genomospecies 8 *sensu* Gardan et al. [7] using MLST analysis and housekeeping genes, and we found that *Psa* is phylogenetically closer to *P. s.* pv. *theae* than to *P. avellanae*. Our analysis, performed with all nine genomospecies (except genomospecies 7) currently circumscribes the majority of the *P. syringae* pathovars and related phytopathogenic pseudomonads and also confirms that the genomospecies 3, including *Pto*, is the most closely-related cluster. The very high genetic similarity between *Psa* and *P. s*. pv. *theae*, a phytopathogen so far reported solely in Japan, reinforces the assumption that *Psa* might be of Asian origin. In fact, there are several reports on the occurrence of this pathogen isolated from both *A. chinensis* and *A. deliciosa* in China [15], [16], [20], [21], South Korea [11], [13], [22] and from *A. deliciosa* and wild *A. arguta* and *A. kolomikta* plants in Japan [10], [23], [24]. The possibility of an Asian origin has been already argued [23].

However, some of the genetic features found in the *Psa* strains of the past and recent epidemic in Italy and New Zealand indicate that different evolutionary routes have been followed by the ancestor(s) of the two different *Psa* populations which are represented by J-*Psa*/I-*Psa* and by I2-*Psa*. Our genome-wide analysis indicated that the two strains J-*Psa* and I-*Psa*, which were isolated in different years from the same kiwigreen cultivar, in geographically distant areas that were affected by outbreaks of bacterial canker of different severity, are extremely similar. This evidence indicates the major role of climatic conditions on the epidemic of bacterial canker to *A. deliciosa*. Furthermore, our comparative study of *Psa* strains shows that despite the apparent prevalence worldwide of a clonal population, a reservoir of diversity of the pathogen has been maintained, which has allowed for a new population, represented by I2-*Psa*, to emerge about 25 years later from an independent evolutionary line with different genetic characteristics and enhanced epidemic potential. Bacterial canker caused by *Psa* to both *A. chinensis* and *A. deliciosa* has also been recently reported in New Zealand. The molecular typing performed using the MLST analysis of seven housekeeping genes and the detection of 12 effector protein genes allowed to ascertain that the *Psa* strains of the current epidemics in Italy, here represented by I2-*Psa*, are identical to those highly virulent strains found in New Zealand, which also lack of the phaseolotoxin gene cluster [27]. Notably, in that country, another *Psa* population, genetically different and less virulent (i.e. apparently causing only leaf spots) than I2-*Psa* but capable to infect both kiwigreen and kiwigold has also been identified. Such a population also differs from the two other genetically different *Psa* populations of the past outbreaks of bacterial canker on *A. deliciosa* in Asia (i.e. Japan and South Korea) and has been retained as endemically in New Zealand [27]. Whether the highly virulent *Psa* population currently causing severe economic losses to *A. chinensis* and *A. deliciosa* in Italy and New Zealand originated in the area of the origin of the pathogen or from the less virulent endemic *Psa* population recently isolated in New Zealand still remain to be verified. However, during the last 30 years, four genetically distinct *Psa* populations have infected, to different extents, different *Actinidia* species on different continents which is a remarkable case of multiple convergent evolution of phytopathogenic pseudomonad populations of the same pathovar to one single plant genus.

We cannot establish with certainty the origin of this re-emerging wave of bacterial canker in Italy although a likely scenario could be hypothesised. The past outbreaks only caused leaf spot and twig die-back but never the death of thousands of plants. The current I2-*Psa* population could have been introduced from abroad through latently infected propagative material or infected pollen. Once established in central Italy, it further reached the other Italian regions by means of latently infected propagative material.

We also found, by assessing the *Psa* growth in the leaves, that *A. deliciosa* cv. Hayward was more susceptible than *A. chinensis* cv. Hort16A when inoculated with the *Psa* strains causing past outbreaks of bacterial canker in Japan and Italy. By contrast, I2-*Psa* multiplication trend was higher in *A. chinensis*. However, a relevant inoculum was also found in *A. deliciosa* three weeks after the inoculation, thus confirming that the re-emerging wave of bacterial canker has been caused by a *Psa* population that has a high fitness for both *Actinidia* species. In the Latium region (central Italy), *A. chinensis* probably largely contributed to the very rapid expansion of such population in the area of kiwigold cultivation, which also acted as reservoir of infection for *A. deliciosa.*

The question then arises as to how this new highly virulent *Psa* population originated. The importance of stress factors in promoting bacterial evolution has been recently pointed out. In fact, under stress condition in the host (i.e. nutrient deficiency outside the host, attack by antimicrobial compounds inside the host, and low temperatures), the bacterial competency to uptake DNA is activated and the pathogen can acquire exogenous genetic material that could help it to escape from the stress [61]. In addition, a possible loss of mobile genetic elements carrying avirulence genes can lead to an enhanced virulence [62]. I2-*Psa* does not have the 50 kb plasmid and the phaseolotoxin gene cluster present in J-*Psa* and I-*Psa* but has gained a 160 kb plasmid and a putative prophage that does not occur in the other population. It remains to be verified if any of these stress factors could have promoted the rise of such new *Psa* population. We did not investigated in details the structure of plasmids but, similarly to other *P. syringae* pathovars, the three *Psa* strains have the *repA* gene that is retained essential for the replication of the pPT23A-like plasmid family [63].

One of the more striking pieces of evidence of the difference between the two populations is the presence of the phaseolotoxin gene cluster, *argK-tox*, in J-*Psa* and I-*Psa* and its absence in I2-*Psa* and in all the other strains of the current epidemic assessed so far in Italy and New Zealand [19], [27]. Phaseolotoxin is considered a major virulence factor for both *Psa* and *Pph* [21], [64]. The *argK-tox* gene cluster is located on the chromosome and was supposedly acquired through lateral gene transfer from bacteria distantly related to *P. syringae* or from non pathogenic or avirulent *P. syringae* strains [54], [55]. Additionally, one *Psy* strain, which was isolated from *Vicia sativa*, displays such a gene cluster [65], although it showed a nucleotide identity of only 85,3% with that displayed by *Psa* and *Pph* [66]. The presence of the *argK-tox* cluster in one variable region of the J-*Psa* and I-*Psa* genomes confirms the acquisition of such genetic trait by lateral gene transfer. Also within *Pph* have been found many pathogenic strains lacking the phaseolotoxin cluster but very aggressive, similarly to I2-*Psa*, towards their host plant [67]. Tamura et al. [29], using an *argK-tox^-^* mutant of KW11 (J-*Psa*) found that the bacterium induced the same type of symptoms in the plant similar to the wild-type, except for the chlorotic halo surrounding the leaf spots. However, the toxin did not promote bacterial growth *in planta*. It has been postulated that phaseolotoxin, by inhibiting ornitine-carbamoxyltransferase, can reduce or inhibit the growth of other microorganisms [64]. The high identity of the *argK-tox* clusters found in strains of *Psa* and *Pph* suggests a recent acquisition of the cluster by the two pathovars [66]. The worldwide occurrence of many highly virulent strains lacking the *argK-tox* gene cluster in both *Psa* and *Pph* allows us to speculate that the ancestral genomes of these two phytopathogens did not include the phaseolotoxin gene cluster.

Other differences between the two *Psa* populations were found by the assessment of the effector protein genes. The three strains display an identical core repertoire of 33 *hop* and 6 *avr* effector genes. However, the *Psa* strains also possess some unique effector protein genes. *HopX1, hopAR*, *hopBD2* and *hopPmaB* are unique to J-*Psa* and I-*Psa*, whereas *hopA1, hopAA1-2, hopH1*, and *hopZ2-*like are unique to I2-*Psa*. Such a different effector repertoire found in the three *Psa* strains might account for the differential aggressiveness showed by the pathogen to *Actinidia* species. However we still do not know the roles played by these effector genes in terms of pathogenicity and host range for the pathovar *actinidiae*. In a recent extensive study regarding the evolution of pathogenicity within the *P. syringae* complex, Baltrus et al. [68] sequenced also a *Psa* strain, namely MAFF302091, isolated from *A. deliciosa* in Japan, in 1984 in the Kanegawa district. Interestingly, this strain differs from J-*Psa*, isolated in Shizuoka district, for the presence/absence of some effector genes. This would indicate that each single strains might possess a distinct effector repertoire. In a comparative study on the occurrence of effector genes in many *P. syringae* strains, also Sarkar et al. [69] analysed *Psa* MAFF302091. Similar to J-*Psa*, also *Psa* MAFF302091 also does not contain *hopA1* and *hopAA1-2*. Noteworthy, these two effectors are rarely found contemporarily present in the 91 *P. syringae* strains tested and only three *Pto* strains showed such effectors in their repertoire [69]. However, our pathogenicity test showed that the three *Psa s*trains did not cause infection to tomato. Vanneste et al. [70] claimed that *hopA1* was also present in I-*Psa* but, according to the genome sequencing, their results appear as an artifact.

Phages, prophages and their morons (i.e. DNA elements inserted between a pair of genes in one phage genome) are known to shape the pathogenicity and virulence of bacterial pathogens and their presence within the bacterial genome can largely contribute to the genetic and phenotypic diversity of bacteria and to the emergence of pathogenic variants [71]. In fact, by carrying various elements contributing to virulence, prophages can also contribute to the individuality of bacteria strains as found in *Salmonella*, *Lactobacillus* and *Burkholderia* [71], [72], [73]. In *Xylella fastidiosa*, the causative agent of infectious diseases of many cultivated crops, prophage-associated chromosomal rearrangements and deletions have been found to be largely responsible for strain-specific differences [74]. Interestingly, in a variable region of I2-*Psa,* we found the presence of many putative proteins related to the assembly and acquisition of prophage PSPPHO6, which are also present in *Pph* 1448A. It remains to be ascertained if there is a link between the relevant virulence of I2-*Psa* to *Actinidia* spp. and the presence of this prophage which was acquired by horizontal gene transfer and if it could have contributed to the further adaptation of *Psa* to *Actinidia* spp.

It is interesting to observe that all three *Psa* strains sequenced here were isolated from leaf spot symptoms but display a set of genes involved in the degradation of lignin derivatives and other phenolics. In fact, similarly to other *P. syringae* pathovars associated with woody hosts such as *P. syringae* pv. *aesculi* and *P. savastanoi* pv. *savastanoi* and to *P. putida*, a soil-inhabiting species [39], [40], the three *Psa* strains have genes putatively related to the degradation of the anthranilate to the cathecol branch of the β-ketoadipate pathway and to the protocatechuate degradation via the protocathechuate 4,5-dioxygenase pathway. These pathways allow for the utilisation of unsubstituted lignin-related compounds and other plant derived phenolic compounds such as mandalate and phenol [75]. This could explain one of the most striking symptoms induced by *Psa* on *Actinidia* spp., the extensive degradation of the woody tissues of the main trunk and leaders mainly occurring during winter. In Italy, I2-*Psa* incites canker of larger dimensions on *A. chinensis* cultivars compared to *A. deliciosa* and sometimes also causes the complete destruction of all external woody tissues, as shown in Figure 1c. It is also worth noting that I2-*Psa* survived more than 45 days in infected *A. chinensis* twigs that were pruned and subsequently brought into the lab, without receiving any amendment (i.e. water) (Marcelletti and Scortichini, unpublished data).

The fact that *Psa* strains can infect both herbaceous (i.e. leaves and young twigs) and woody tissues of the same host plant could mean that differential set of genes are activated when the pathogen is multiplying and infecting different organs of the plant. It would be interesting to further investigate the *P. syringae* complex to determine whether the capability of infecting herbaceous tissues appeared before or after that of infecting woody tissues.

All *Psa* strains display also sets of genes that are virulence factors or are important for the survival of the bacterium *in planta* or for competing with other micro-organisms. In fact, *Psa* has genes involved in the inhibition of nitric oxide metabolism, namely nitric oxide dioxygenase and anaerobic nitric oxide reduction [76]. Nitric oxide plays a fundamental role in plant disease resistance by acting as a signal-inducing plant gene to synthesise defense-related compounds [77]. The inhibition of nitric oxide synthesis consequently promotes the bacterial growth *in planta*.

*Psa* strains contain *copA* and *copB*, genes that play a key role in copper resistance [56]. Copper ions are essential for bacterial species but can incite toxic cellular effects if levels of free ions are not controlled. It has been observed that strains of *P. syringae* with no known history of exposure to copper selection accumulate copper and are resistant. In these bacteria, copper accumulation may have a beneficial role other than in resisting high levels of copper [56]. Interestingly, Nakajima et al. [57] found that at the beginning of bacterial canker outbreaks in Japan (i.e. 1984–1987) all the *Psa* strains displayed solely *copA* and *copB*. However, after repeated spray treatments with copper-based bactericides, the *Psa* strains also showed additional genes responsible for the maximal resistance to copper, namely *copR* and *copS.*

*Psa* can counteract the lethal effect of antibiotics by means of multidrug efflux pumps of the multidrug resistance systems encoded by chromosomal genes. These features apparently confer a relevant fitness for the *in planta* growth of *Psa.* Notably, I2-*Psa* also displays a lantibiotic dehydratase protein that might putatively inactivates this class of antibiotics which is produced by Gram-positive bacteria and characterised by its high specific activity against multidrug-resistant bacteria [78].

The efficient uptake and utilisation of iron through siderophores is regarded as an important virulence factor for phytopathogenic pseudomonads, especially in iron-limited environments [79]. The *Psa* strains have a set of genes coding for the production of siderophores such as pyoverdine, haemin, enterobactin and yersiniabactin. These last two siderophores are primarily described in the *Enterobacteriaceae*, and they are characterized by a very high affinity for iron [80], [81]. Similar to *P. s.* pv. *aesculi*, *Psa* strains also contain hemagglutinin-like proteins. Although they have not been investigated in pseudomonads, in other phytopathogenic bacteria such as *Erwinia chrysanthemi, Xylella fastidiosa* and *Xanthomonas oryzae* pv. *oryzae* these protein have been shown to specifically act in adhesion between the bacterial cell and the plant host cell [82], [83].

One laboratory claimed that *Psa* NCPPB3871 (I-*Psa*) which was directly received from the National Collection of Plant Pathogenic Bacteria, is not to be a genuine *Psa* strain [84]. We and other labs [66] did not find such discrepancy. A putative contamination in the former lab could have occurred.

The comparative genome-wide analysis performed with these three *Psa* strains representing two different populations of the pathovar, provides important insights into the evolution and adaptation of this pathogen to *Actinidia* spp. and highlights how a virulence factor like the phaseolotoxin can be lost without decreasing the relative virulence of the bacterium. We also demonstrate how the mobile arsenal of phytopathogenic bacteria (i.e. plasmids and prophages) can be lost and gained by populations of the same pathovar that, consequently, can modulate their virulence accordingly.

## Materials and Methods

### Bacterial strains

*Psa* NCPPB3739 is the type strain of the pathovar and was isolated in 1984, in Japan (Shizuoka district) from a leaf spot lesion of *A. deliciosa* cv. Hayward [10]. *Psa* NCPPB3871 was isolated in 1992 in the Roma province (central Italy) from a leaf spot lesion on *A. deliciosa* cv. Hayward [12]. *Psa* CRA-FRU 8.43 was isolated in the province of Latina (central Italy) from a leaf spot lesion of *A. chinensis* cv. Hort16A [14], [19], and is the first isolate from the epidemic of bacterial canker currently causing severe damage to the cultivation of *A. chinensis* and *A. deliciosa* in Italy [25]. In this paper, the *Psa* strains are referred to as J-*Psa* (NCPPB3739), I*-Psa* (NCPPB3871) and I2-*Psa* (CRA-FRU 8.43). Figure 1 shows the symptomatic leaves of *A. deliciosa* and *A. chinensis* in Italy from where isolations were performed. For this study, the *Psa* strains were maintained on nutrient agar amended with 5% w/v sucrose (NSA) and incubated at 25±1°C:

### Library preparation and Illumina sequencing

Bacterial genomic DNA was extracted from 1 ml of overnight J-*Psa*, I-*Psa* and I2-*Psa* cultures grown in KB broth DNA using a Wizard DNA purification kit (Promega Italia, Padova, Italy) following the manufacturer's instructions. DNA was measured and checked for quality using a NanoDrop (NanoDrop products, Wilmington, DE, USA). A total of 10 µg of DNA from each sample was fragmented by incubation for 70 min with 5 µl of dsDNA Fragmentase (New England Biolabs MA, USA). The reaction was stopped with EDTA and purified using a QIAquick PCR purification kit (QIAGEN, Hilden, Germany). The eluate was end repaired using an End Repair kit (New England Biolabs, MA, USA) for 30 min at 20°C. The end-repaired DNA was A-tailed for 30 min at 37°C using a d-A Tailing kit (New England Biolabs, MA, USA). After purification using the MinElute purification kit (QIAGEN), the DNA was ligated using Quick T4 DNA ligase (New England Biolabs) to 500 pmol of Illumina adaptors that had been previously annealed by heating at 98°C for 3 min and then slowly cooling to 16°C in a thermocycler. After further purification using the MinElute purification kit (QIAGEN), 1 µl of each reaction was quantified by labelling with biotin, spotted on nitrocellulose after a serial dilution, and detected using an anti-biotin-AP conjugate (Roche Diagnostics, Monza, Italy) following manufacturer's instructions. Equal amounts of DNA from samples were pooled together and size fractionated by 2% MS-6 agarose (Conda, Madrid, Spain) gel electrophoresis in TAE buffer at 120 V for 60 min. Gel slices containing DNA in the 400 to 600 bp estimated range were cut and purified using QIAquick gel extraction kit (QIAGEN) and used for sample preparation according to the protocol for genomic DNA sequencing using the Illumina Genome Analyser IIx (Illumina, USA). The samples were run at the Istituto di Genomica Applicata (Udine, Italy).

The Illumina sequencing provided complessively nearly 10 millions 100 nt reads from the genomic DNA of the three *Psa* strains that passed the quality check. This amount of sequence represents approximately 27.7, 67.6 and 61.2 X coverage for J-*Psa*, I-*Psa* and I2-*Psa*, respectively, i.e. the expected genomic size of these strains, based on the previously sequenced genomes of the *P. syringae* pathovars is 6 Mb.

### Whole-genome assembly and alignment of Illumina genomes

Paired reads of 100 nts were assembled into contigs using the *de novo* (*i.e.* without using a reference genome) assembly option of the CLC genomic workbench (CLC-bio, Aarhus, Denmark). Contigs sequences were scanned for ORFs by GLIMMER, version 3.02. [85] which had been previously trained on the complete genome sequences of *Pseudomonas syringae* pv. *tomato* strain DC3000 (NC_004578.1, i.e. *Pto* DC3000), *P. s.* pv. *phaseolicola* strain 1448A (NC_005773.3, *i.e*. *Pph* 1448A), and *P. s.* pv. *syringae* strain B728a (NC_007005.1, *i.e*. *Psy* B728a). The putative proteins were annotated against the RefSeq database using a perl script for recursive blastx searches. Additional genome sequence analyses was performed with the aid of the software packages mummer [86] and mauve [87]. Several *ad hoc* PERL scripts were developed to assist the comparison of genome sequence draft and its putative protein complement with respect to each one of the three *Psa* strains and *Pto* DC3000, *Pph* 1449A and *Psy* B728a. Additional assessment of the effector genes repertoire was performed upon the paper of Baltrus et al. [68]

### Protein categorization and searching for variable regions in *Psa* genomes

For each strain, all predicted proteins were ordered by role categories according to TIGRFAMs. In addition, for each *Psa* strain the presence along the genomes of variable regions was also analysed. Such genomic regions were identified by the following criteria using MAUVE and an *ad hoc* script PERL “island finder” a) DNA region larger than 10 kb in a contig that appeared as a gap in the genome alignment and b) different G+C content with respect to the average content of the three *Psa* strains, which was performed using the tool: http://www.sciencebuddies.org/science-fair-projects/projectideas/GenomGCcalculator. These regions were retained as good candidates to be the results of horizontal gene transfer [88].

### Phylogeny

All the sequences used in the MLST analysis to build the phylogenetic trees were edited and aligned using ClustalW 1.83 (http://www.ebi.ac.uk/tools/clustalw2/) and concatenated using Geneious 5.1.4 (http://www.geneious.com/). The phylogenetic tree built using the neighbor-joining (NJ) algorithm was obtained using the Splits-Tree software [89] and the Hamming distances method using three housekeeping gene fragments, namely *gyrB, rpoB* and *rpo,* for a total of 1,646 bp. Bootstrap analysis was performed using the same software. The program TOPALi version 2.5, available at (http://www.topali.org/) [90], was used to determine the best model of evolution for the second phylogenetic tree based on maximum likelihood (ML) algorithm using six housekeeping fragment genes, *acnB, fruK, gltA, pgi, rpoB* and *rpoD,* for a total of 2,926 bp. The PhyML [91] method was used to determine the best model of evolution for the ML analysis. Both the hierarchical likelihood ratio test (hLRT) and the standard Akaike Information Criterion (AIC) were used to evaluate the model scores. Bootstrap analysis was performed using the same software. The hypotheses about the genealogy of the *Psa* strains were tested using the likelihood-ratio test for monophyly which was developed by Huelsenbeck et al. [92]. Likelihoods were estimated using the Phangorn module [93] of the statistical package R (R development core team, 2007). The significance of the likelihood ratio was estimated by parametric bootstrap according to Huelsenbeck et al. [92] by simulation of 1,000 replicated datasets generated with Indel-Seq-Gen [94].

### Analysis of plasmid content

Plasmid isolation was performed using the PureYield^TM^ Plasmid Miniprep System (Promega, Madison, WI, U.S.A.) protocol. The strains submitted for plasmid detection were the object of this study as well as *Psa* KW30 and *Psa* KW31, which were isolated in 1984 in Japan from leaf spot lesions of *A. deliciosa* cv. Hayward [10] and additional 11 *Psa* strains isolated in different regions of Italy during current epidemics [14], [25], [42]. Plasmids from *Escherichia coli* strain 39R861 were used as molecular weight marker [95].

### Multiplication of *Psa* in *Actinidia* spp. leaves and pathogencity test on tomato

To compare the capability of infection in different *Actinidia* spp., we artificially inoculated both *A. chinensis* and *A. deliciosa* leaves with all three of the sequenced *Psa* strains. For inoculation, bacteria were grown for 48 h on NSA, at 25±1°C, and the plants were covered 24 h before the inoculation with a plastic bag. Leaf areas of approximately 1 cm in diameter on one-year-old potted *A. deliciosa* cv Hayward and *A. chinensis* cv Hort16A plants were inoculated using a needleless sterile syringe with a bacterial suspension in sterile saline (0,85% NaCl in distilled water) at the concentrations of 1–2×10^3^ and 1–2×10^6^ cfu/mL. For each thesis, 10 leaves were inoculated in four sites. Control plants were treated using solely sterile saline. To determine bacterial growth *in planta*, leaf disks of about 0,5 cm of diameter were sampled from each species and inoculation site at regular intervals and ground in 1 mL of sterile saline, and serial ten-fold dilutions were spotted onto NSA medium. Colonies were counted two days after incubation at 25±1°C. According to a comparative study on host-specific virulence factors and effector genes [54], it has been observed that the contemporary presence of the effector genes *hopAA1-2* and *hopA1* in single strains is quite rare. Only three *Pto* strains displayed such a combination. To verify that *Psa,* in particular I2-*Psa* showing such effector genes, can multiply and also infect tomato plants, we performed a pathogenicity test by inoculating potted-seedlings of *Lycopersicon esculentum* (tomato) cv Lancelot. The inoculation was performed as described above and *Pto* DC3000 was used as positive control. Symptoms caused by inoculation of the three *Psa* strains were observed and compared with those caused by *Pto* DC3000.

## Supporting Information

Figure S1 Evolutionary relationships of *Psa* strains to other phytopathogenic pseudomonads. Phylogenetic relationships were estimated from concatenated sequences from six housekeeping genes, *acnB, fruK, gltA, pgi, rpoB* and *rpoD* (2,926 bp), using the maximum likelihood (ML) algorithm. Bootstrap values are reported at each branching. *P. putida* was used as outgroup.
(DOC)

Figure S2 Presence and absence of phaseolotoxin in *Psa* strains. Diagrammatic representation of the phaseolotoxin gene cluster, *argK-tox*, and the flanking regions. The phaseolotoxin cluster is present in J*-Psa* and I-*Psa* (upper part) but not in I2-*Psa* (lower part).
(DOC)

Table S1 Variable regions (VR) found in the draft genome of I2-*Psa* compared with J-*Psa* and I-*Psa* draft genomes.
(DOCX)

Table S2 Categorization according to TIGRFAMs of protein complement displayed by the draft genome of I2-*Psa*.
(XLS)

Table S3 Similarity of the type III effector protein genes complement of J-*Psa*, I-*Psa* and I2-*Psa* compared with the same complement of other sequences plant pathogenic pseudomonads.
(XLS)
