# Supplementary figures and images for: Pseudomonas syringae pv. actinidiae Draft Genomes Comparison Reveal Strain-Specific Features Involved in Adaptation and Virulence to Actinidia Species
Source: PLoS One. 2011 Nov 23;6(11):e27297. doi: 10.1371/journal.pone.0027297 (PMC3223175; doi:10.1371/journal.pone.0027297)

Figure S1.


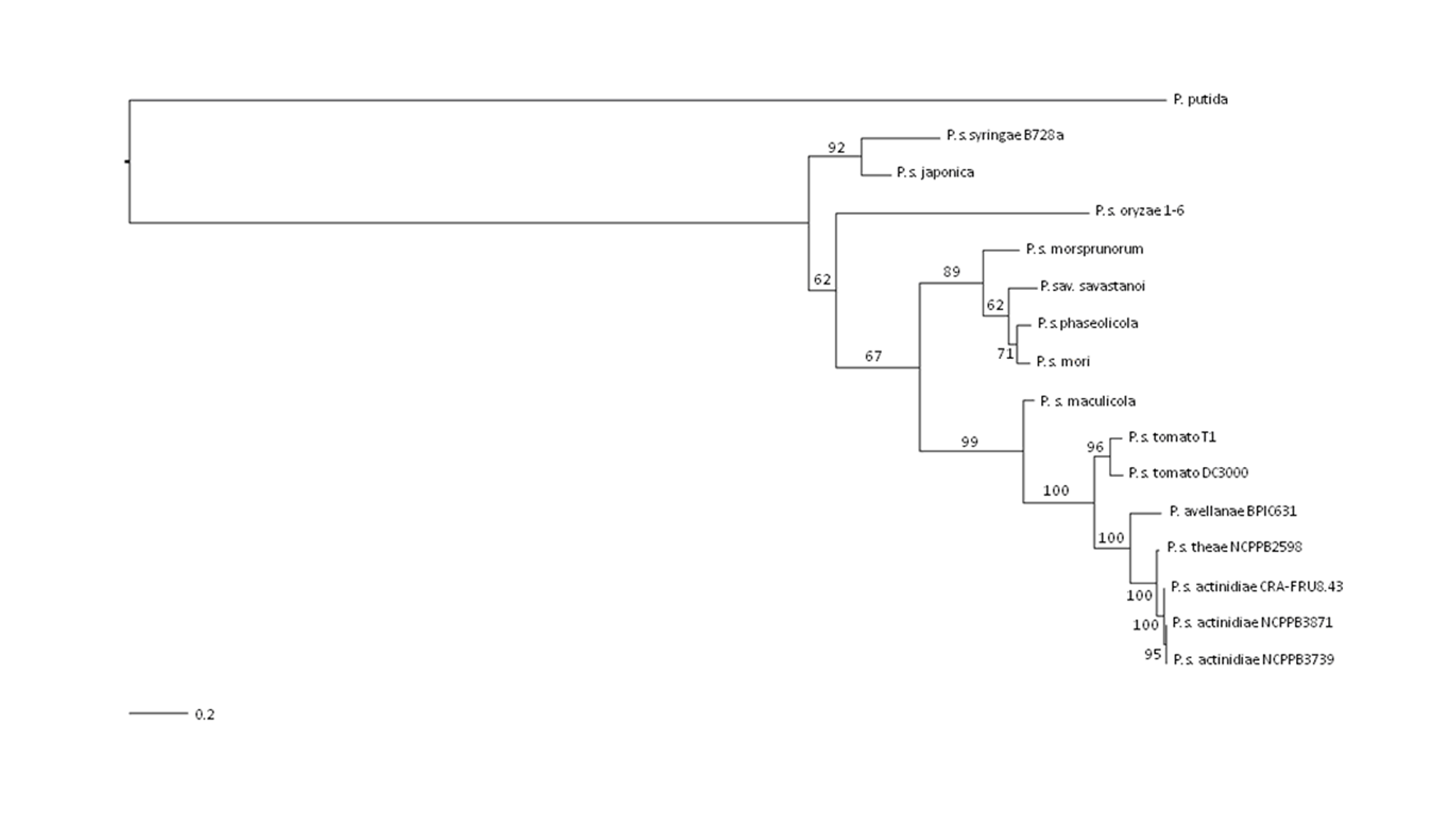

Supplement: Figure S1 — Evolutionary relationships of Psa strains to other phytopathogenic pseudomonads. Phylogenetic relationships were estimated from concatenated sequences from six housekeeping genes, acnB, fruK, gltA, pgi, rpoB and rpoD (2,926 bp), using the maximum likelihood (ML) algorithm. Bootstrap values are reported at each branching. P. putida was used as outgroup. (DOC) [file pone.0027297.s001.doc]

Figure S2.


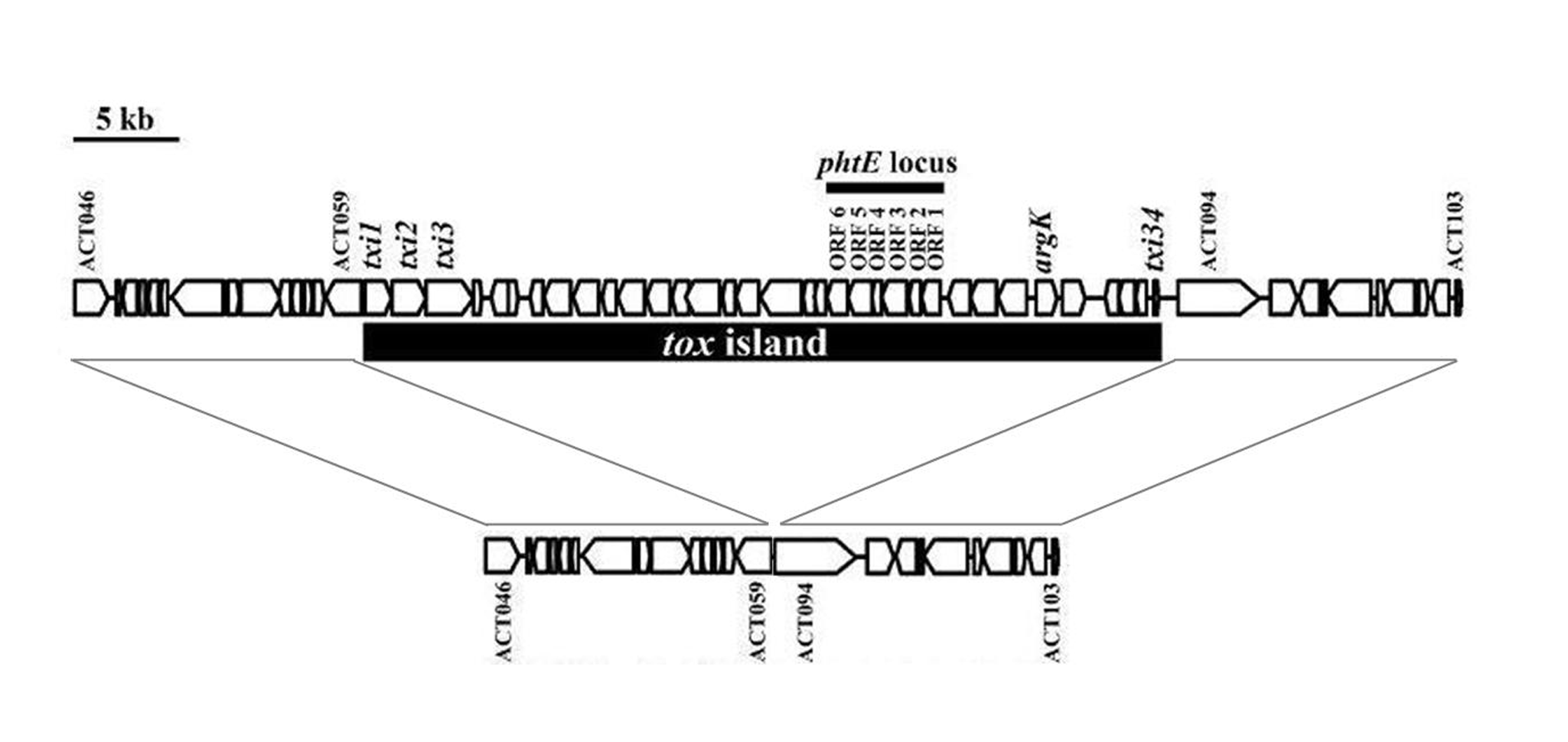

Supplement: Figure S2 — Presence and absence of phaseolotoxin in Psa strains. Diagrammatic representation of the phaseolotoxin gene cluster, argK-tox, and the flanking regions. The phaseolotoxin cluster is present in J-Psa and I-Psa (upper part) but not in I2-Psa (lower part). (DOC) [file pone.0027297.s002.doc]
