# Supplementary material for: Pseudomonas syringae pv. actinidiae Draft Genomes Comparison Reveal Strain-Specific Features Involved in Adaptation and Virulence to Actinidia Species
Source: PLoS One. 2011 Nov 23;6(11):e27297. doi: 10.1371/journal.pone.0027297 (PMC3223175; doi:10.1371/journal.pone.0027297)
Supplement: Table S1 — Variable regions (VR) found in the draft genome of I2-Psa compared with J-Psa and I-Psa draft genomes. (DOCX) [file pone.0027297.s003.docx]

Table S1.

| **VR** | **Strains** | **Contigs** | **Coordinate** | **% GC** | **VR-encoded genes** |
| --- | --- | --- | --- | --- | --- |
|  |  |  |  |  |  |
| 1 | CRA-FRU8.43A | Contig50 | from 25970 to end | 53.6 | 16 Hypothetical protein |
|  |  |  |  |  | 6 No database match |
|  |  |  |  |  | 2 Histidine kinase |
|  |  |  |  |  | C4-dicarboxylate transporter |
|  |  |  |  |  | Conserved hypothetical protein |
|  |  |  |  |  | DNA topoisomerase III |
|  |  |  |  |  | Inorganic pyrophosphatase |
|  |  |  |  |  | Orf28 |
|  |  |  |  |  | Phage integrase |
|  |  |  |  |  | Phosphopyruvate hydratase |
|  |  |  |  |  | Putative transporter |
|  |  |  |  |  | Regulatory protein, LysR:LysR, substrate-binding |
|  |  |  |  |  | Site-specific recombinase, phage integrase family |
|  |  |  |  |  | Voltage-gated chloride channel family protein |
|  |  |  |  |  |  |
|  |  |  |  |  |  |
|  |  |  |  |  |  |
| 2 | CRA-FRU8.43A | Contig 304 | from 174 to 10544 | 53.3 | 2 Hypothetical protein |
|  |  |  |  |  | 2 No database match |
|  |  |  |  |  | NERD domain protein |
|  |  |  |  |  |  |
|  |  |  |  |  |  |
|  |  |  |  |  |  |
| 3 | CRA-FRU8.43A | Contig 304 | from 11917 to end | 54.8 | 3 Hypothetical protein |
|  |  |  |  |  | 2 No database match |
|  |  |  |  |  | 2 Transposase component |
|  |  |  |  |  | Conserved hypothetical protein |
|  |  |  |  |  | Putative transposase |
|  |  |  |  |  | RecF/RecN/SMC N domain protein |
|  |  |  |  |  | UvrD/REP helicase |
|  |  |  |  |  |  |
|  |  |  |  |  |  |
|  |  |  |  |  |  |
| 4 | CRA-FRU8.43A | Contig 110 | from start to 12623 | 53.8 | 9 Hypothetical protein |
|  |  |  |  |  | P-loop ATPase-like protein |
|  |  |  |  |  | TraG family conjugative protein |
|  |  |  |  |  |  |
|  |  |  |  |  |  |
|  |  |  |  |  |  |
| 5 | CRA-FRU8.43A | Contig 110 | from 28282 to 43193 | 53.2 | 4 Hypothetical protein |
|  |  |  |  |  | 2 No database match |
|  |  |  |  |  | ArsH protein |
|  |  |  |  |  |  |
|  |  |  |  |  |  |
|  |  |  |  |  |  |
| 6 | CRA-FRU8.43A | Contig 37 | from start to 18097 | 55.8 | 3 Hypothetical protein |
|  |  |  |  |  | 2 EAL domain-containing protein |
|  |  |  |  |  | 2 No database match |
|  |  |  |  |  | 2 Response regulator/sensor histidine kinase |
|  |  |  |  |  | CDP-diacylglycerol--glycerol-3-phosphate 3-phosphatidyltransferase |
|  |  |  |  |  | DNA-binding response regulator GacA |
|  |  |  |  |  | Excinuclease ABC, C subunit |
|  |  |  |  |  | Fimbrial protein |
|  |  |  |  |  | Fimbrial usher protein |
|  |  |  |  |  | Gram-negative pili assembly chaperone |
|  |  |  |  |  | LuxR family DNA-binding response regulator |
|  |  |  |  |  | pili assembly chaperone |
|  |  |  |  |  | Putative DNA-binding protein |
|  |  |  |  |  |  |
|  |  |  |  |  |  |
|  |  |  |  |  |  |
|  |  |  |  |  |  |
| 7 | CRA-FRU8.43A | Contig 101 | from 2713 to end | 59.1 | 13 Hypothetical protein |
|  |  |  |  |  | 2 Prophage PSPPH06, putative tail fiber protein |
|  |  |  |  |  | Gp5 domain-containing protein |
|  |  |  |  |  | Prophage PSPPH06, DksA/TraR family C4-type zinc finger protein |
|  |  |  |  |  | Prophage PSPPH06, lysis protein |
|  |  |  |  |  | Prophage PSPPH06, putative head completion/stabilization protein |
|  |  |  |  |  | Prophage PSPPH06, putative lysozyme |
|  |  |  |  |  | Prophage PSPPH06, putative tail protein |
|  |  |  |  |  | Prophage PSPPH06, putative tail sheath protein |
|  |  |  |  |  | Prophage PSPPH06, putative tail tube protein |
|  |  |  |  |  | Prophage PSPPH06, TP901 family tail tape measure protein |
|  |  |  |  |  | Prophage PSPPH06, virion morphogenesis protein |
|  |  |  |  |  |  |
|  |  |  |  |  |  |
|  |  |  |  |  |  |
| 8 | CRA-FRU8.43A | Contig 84 | start to 27218 | 51.8 | 4 Hypothetical protein |
|  |  |  |  |  | 2 No database |
|  |  |  |  |  | CiaB protein |
|  |  |  |  |  | Conserved hypothetical protein |
|  |  |  |  |  | N-6 DNA methylase |
|  |  |  |  |  | Putative helicase |
|  |  |  |  |  | Putative ParB-like protein |
|  |  |  |  |  | Putative plasmid partitioning protein |
|  |  |  |  |  | Putative recombinase |
|  |  |  |  |  | Restriction modification system DNA specificity domain protein |
|  |  |  |  |  | Type I restriction enzyme (modification subunit) |
|  |  |  |  |  | Transposase |
|  |  |  |  |  | Transposition helper protein |
|  |  |  |  |  |  |
|  |  |  |  |  |  |
|  |  |  |  |  |  |
| 9 | CRA-FRU8.43A | Contig 188 | start to end | 53.4 | 2 Hypothetical protein |
|  |  |  |  |  | Amino acid adenylation |
|  |  |  |  |  | Amino acid adenylation domain-containing protein |
|  |  |  |  |  | Aminotransferase class-III |
|  |  |  |  |  | Arabinose efflux permease family protein |
|  |  |  |  |  | FMN-dependent alpha-hydroxy acid dehydrogenase |
|  |  |  |  |  | Lantibiotic dehydratase domain-containing protein |
|  |  |  |  |  | MbtH-family protein |
|  |  |  |  |  | Non-ribosomal peptide synthetase |
|  |  |  |  |  | Putative non-ribosomal peptide synthetase |
|  |  |  |  |  | Putative transcriptional regulator, GntR family |
|  |  |  |  |  | 4-hydroxyphenylpyruvate dioxygenase |
|  |  |  |  |  |  |
|  |  |  |  |  |  |
|  |  |  |  |  |  |
| 10 | CRA-FRU8.43A | Contig 104 | start to end | 55.3 | 4 Hypothetical protein |
|  |  |  |  |  | 2 Putative DNA repair protein ruvB |
|  |  |  |  |  | No database match |
|  |  |  |  |  | SMC domain-containing protein |
|  |  |  |  |  |  |
|  |  |  |  |  |  |
|  |  |  |  |  |  |
| 11 | CRA-FRU8.43A | Contig 263 | start to end | 57.1 | 6 Hypothetical protein |
|  |  |  |  |  | DNA primase-like protein |
|  |  |  |  |  | No database match |
|  |  |  |  |  | PBSX family phage portal protein |
|  |  |  |  |  | Phage protein |
|  |  |  |  |  | Prophage PSPPH06, major capsid protein P2 family |
|  |  |  |  |  | Putative terminase, endonuclease subunit |
|  |  |  |  |  | Scaffold protein |
|  |  |  |  |  |  |
|  |  |  |  |  |  |
|  |  |  |  |  |  |
| 12 | CRA-FRU8.43A | Contig 409 | start to end | 54.1 | 4 Hypothetical protein |
|  |  |  |  |  | KAP P-loop |
